# Cannabidiol: potential in treatment of neurological diseases, flax as a possible natural source of cannabidiol

**DOI:** 10.3389/fncel.2023.1131653

**Published:** 2023-04-17

**Authors:** Maksim V. Storozhuk

**Affiliations:** Department of Cellular Membranology, Bogomoletz Institute of Physiology, National Academy of Sciences of Ukraine, Kyiv, Ukraine

**Keywords:** cannabidiol, neurological diseases, epilepsy, anxiety, anxiolytic, GABA_A_ receptors

Cannabidiol (CBD) is a non-psychoactive phytocannabinoid obtained from Cannabis (Jung et al., 2019). As a part of Cannabis-based medicinal preparation, CBD has been used for a long time; however, until recently, it has received far less attention as a single drug (Morales et al., 2017). Currently, CBD and its analogs receive growing attention because of their potential therapeutic benefits (Pisanti et al., 2017; Jung et al., 2019), including neuroprotective (Campos et al., 2016), anti-epileptic (Silvestro et al., 2019), anti-inflammatory (Couch et al., 2017), anxiolytic (Lee et al., 2017), and anti-cancer (Massi et al., 2013) properties. The clinical use of CBD is the most advanced in the treatment of epilepsy (WHO, 2018). Research in relation to other neurological disorders is substantially less advanced: for most indications, there is only pre-clinical evidence or a combination of pre-clinical and limited clinical evidence (WHO, 2018). Nevertheless, there is growing evidence suggesting the therapeutic potential of CBD for treating several neurological and neuropsychiatric disorders that affect millions of people worldwide (see Vitale et al., 2021; Bhunia et al., 2022 for a recent review).

At the same time, at least in several countries, the use of CBD for medicinal and even for research purposes is complicated or not possible due to legal restrictions that are mostly related to one source of CBD. Indeed, while Cannabis has been recently legalized for medicinal purposes in several countries, it is still illegal for these purposes in many others. Moreover, although the content of the psychoactive cannabinoid tetrahydrocannabinol (Δ9-THC) is substantially lower in hemp, the latter is also illegal for medicinal purposes in some countries. In addition, the usage of synthetic CBD can also be a subject of regulation or unclear (Appendino et al., 2022). Thus, the potential of CBD in the treatment of diseases (defined as the actual possibility of patients using CBD for treatment) is limited by restrictions related to the *source* of this cannabinoid. Indeed, concerns about the source of CBD were a reason for the recent ban (Yeung, 2023) on CBD-containing products in Hong Kong. Considering the above issues taken together, the idea of using CBD from non-cannabis plants seems to be interesting. In this context, the occurrence of cannabinoids in non-cannabis plants with a focus on CBD has recently been reviewed (Appendino et al., 2022). Flax (*Linum usitatissimum*) was considered a reasonable candidate with respect to the occurrence of a CBD-like compound, though levels of this compound were considered very low (Appendino et al., 2022).

In this study, (i) a brief outline of the therapeutic potential of CBD in relation to some neurological diseases and respective molecular targets of CBD is provided, and (ii) some details/concerns regarding flax as a potential natural source of CBD have been discussed with a focus on levels of the CBD-like compound in flax.

## Therapeutic potential of cannabidiol in neurological diseases

The therapeutic potential of CBD in relation to neurological disorders has recently been reviewed in detail in many studies (Vitale et al., 2021; Bhunia et al., 2022). In the following paragraphs, some of these disorders and potential molecular targets of cannabidiol in relation to them are introduced.

*Epilepsy* is a neurological disorder characterized by unprovoked seizures, caused by abnormal brain activity. Epilepsy affects approximately 50 million people worldwide (WHO, 2022). Considering that 30–40% of patients develop drug-resistant epilepsy, the availability of novel treatments is of great importance. Recently, one pure CBD product (Epidiolex) has been approved for patients with rare forms of drug-resistant epilepsy (Vitale et al., 2021). The therapeutic effects of CBD in epilepsy are thought to be mediated by multiple molecular targets, including GABA_A_ receptors, glycine receptors, TRPV1, TRPV2, TRPA1, and GPR55 (Vitale et al., 2021).

*Parkinson's disease* (PD) is a progressive neurodegenerative disorder caused by the degeneration of dopaminergic neurons in the *substantia nigra* of the midbrain and the development of neuronal Lewy bodies (Beitz, 2014). In PD animal models, CBD had neuroprotective effects, probably mediated by its antioxidant and anti-inflammatory properties (Moises Garcia-Arencibia et al., 2007). It was also shown that CBD counteracts non-motor symptoms of PD (Crippa et al., 2019). These works are just two examples see (Vitale et al., 2021; Bhunia et al., 2022) for detailed review. 5-HT3Rs and GPR6 are thought to be molecular targets mediating the therapeutic effects of CBD in PD (Vitale et al., 2021).

*Alzheimer's disease* (AD) is a progressive neurodegenerative disorder, affecting millions of people worldwide. Currently, only symptomatic treatments are available for this disease. Although the accumulation of β-amyloids is considered to be a major histopathological hallmark of AD, it is still debated whether this is the primary *cause* of the disease. Nevertheless, it is generally accepted that multiple factors, including oxidative stress and neuroinflammation, contribute to the progression of AD. Evidence suggesting the potential benefits of CBD in relation to AD has been discussed in detail in a recent review (Bhunia et al., 2022). Here, it will be just mentioned that deficits in hippocampal LTP in a model of AD are reversed by CBD (Hughes and Herron, 2019).

In addition, CBD is potentially useful in numerous other disorders and pathologies that affect the CNS. These include multiple sclerosis, schizophrenia, post-traumatic stress, depression, and anxiety (Campos et al., 2016; Scarante et al., 2020, for a review; see Vitale et al., 2021; Bhunia et al., 2022), and this is just to name a few. In relation to depression and anxiety, CBD has been considered due to its antidepressant- and anxiolytic-like effects observed in animal models (see Schier et al., 2014, for review). Interestingly, some effects of CBD may be gender-dependent (Franzen et al., 2023). Analysis of *clinical trials* suggests that while CBD seems to be effective as an anxiolytic, its efficacy for other non-seizure-related conditions is much more variable (Tang et al., 2022). 5-HT3AR, 5-HT1AR, and the two closely related G-protein coupled receptors, GPR3 and GPR6, are considered as main molecular targets mediating the antidepressive effects of CBD (Vitale et al., 2021). These receptors are also likely to be involved in anxiety disorders (Vitale et al., 2021).

## Flax as a possible natural source of cannabidiol

As already mentioned, the potential of CBD in the treatment of diseases is limited by restrictions related to the source of this cannabinoid. In this context, the presence of a CBD-like compound in fibers and seeds of flax (Styrczewska et al., 2012), with at least several characteristics similar to that of CBD, seems to be important. In brief, while studying the extract from previously generated transgenic plants overproducing phenylpropanoids, Styrczewska et al. found a new terpenoid compound. Further analysis of this compound using UPLC and gaschromatography–mass spectrometry (GC-MS) revealed that this compound is similar to CBD (Styrczewska et al., 2012). In addition, the biological assays carried out on the CBD-like compound showed a similar bioactivity profile of CBD, though the bioactivity assays performed are not specific for CBD. Importantly, *nearly the same* levels of this compound were also found in *wild-type plants* (see Figure 2 in Styrczewska et al., 2012).

In contrast to Cannabis and hemp, the usage of flax is not subject to strict regulation. Indeed, flax is authorized as an addition to foods, in particular, by FDA (Goyal et al., 2014), and is used for the production of several dietary supplements. Thus, given that the CBD compound in flax is indeed CBD, it may be useful in countries with the abovementioned restrictions. Moreover, it should be mentioned that while the results of Styrczewska et al. (2012) are encouraging, further studies on this issue are certainly required. Specifically, the isolation of the CBD-like compound from flax and its firm identification as CBD would be necessary in relation to this issue.

An additional concern is the levels of CBD-like compounds in flax, but these are considered very low (Appendino et al., 2022). However, this issue is not as clear as it may seem. Although the reported levels of CBD in hemp leaves (~800 μg/g, see Knezevic et al., 2021) are definitely much higher than the levels of CBD-like compounds in flax leaves (~7 μg/g, see Styrczewska et al., 2012), strikingly, low levels of CBD were reported in some commercial hemp dietary products such as *dried plant material* (0.64 μg/g) (Meng et al., 2018). The level of the CBD-like compound detected in flax seeds (10 μg/g) is comparable to that in hemp seeds (0.32 to 25.55 μg/g, see Jang et al., 2020). It should be also mentioned that the levels of CBD-like compounds in flax flowers were not determined by Styrczewska et al. (2012). Considering that the levels of CBD in hemp flowers are much higher than in seeds, it cannot be excluded that, similarly, levels of CBD-like compounds in flax flowers are also higher compared to seeds. However, this is not likely because the levels of CBD-like compounds in the seed buds of flax are comparable to those in flax seeds.

In addition, comparing the effects of CBD-like compound from flax with already known effects of CBD in models of neurological diseases may give some interesting results. Just as an example, would CBD-like compound mimic effects of CBD in reversing deficits in hippocampal LTP (Hughes and Herron, 2019)? On the one hand, an answer should hint at whether the biological activity of the CBD-like compound is similar to that of CBD in this pharmacological model. On the other hand, even in the case that a CBD-like compound is not actually CBD, still interesting and potentially useful effects may be found. In any case, given that the compound found in flax is CBD, its levels appear to be sufficient for CBD-containing dietary products.

In summary, CBD is already approved for treating some forms of epilepsy, and there is growing evidence suggesting the therapeutic potential of CBD in relation to many other diseases of the central nervous system. At the same time, at least in several countries, the use of CBD for medicinal and even research purposes is complicated or not possible due to legal restrictions that are mostly related to a source of CBD. In this context, though it is chancy, the results of addressing scientific questions related to the occurrence of CBD-like compounds in flax may have potentially important implications, including implications related to the treatment of diseases of the central nervous system. For instance, given that the CBD-like compound in flax is indeed CBD, it could be used for medicinal properties in countries with legal restrictions on cannabis/hemp. Other potentially useful implications also cannot be excluded. For instance, given that the compound found in flax is CBD, its levels appear to be sufficient for CBD-containing dietary products. At the same time it should be emphasized that the major concern regarding possibility to use flax as a natural source is the exact nature of CBD-like compound in flax. An additional but also important concern is the low levels of CBD-like compounds in flax.

## Author contributions

The author confirms being the sole contributor of this work and has approved it for publication.
